# Study protocol testing toolkit versus usual care for implementation of screening, brief intervention, referral to treatment in hospitals: a phased cluster randomized approach

**DOI:** 10.1186/s13722-018-0130-4

**Published:** 2018-12-27

**Authors:** Robin Newhouse, Michelle Janney, Anne Gilbert, Jon Agley, Giorgos Bakoyannis, Melora Ferren, C. Daniel Mullins, Meg Johantgen, Rhonda Schwindt, Kelli Thoele

**Affiliations:** 1 Indiana University School of Nursing, 600 Barnhill Drive, NU 132, Indianapolis, IN 46202 USA; 2 Indiana University Health, Fairbanks Hall, 340 West 10th Street, Indianapolis, IN 46202 USA; 3 Indiana University Health, Methodist Hospital, 1701 N. Senate Blvd, Indianapolis, IN 46202 USA; 4 Institute for Research on Addictive Behavior, Indiana University School of Public Health - Bloomington, 501 N. Morton Street, Suite 110, Bloomington, IN, 47404 USA; 5 Indiana University Fairbanks School of Public Health and School of Medicine, 410 West 10th Street, Suite 3000, Indianapolis, IN 46202 USA; 6 University of Maryland School of Pharmacy, Saratoga Building, 12th Floor, 220 Arch Street, Baltimore, MD 21201 USA; 7 University of Maryland School of Nursing, 655 W. Lombard Street, Baltimore, MD 21201 USA; 8 The George Washington University School of Nursing, 1919 Pennsylvania Ave. NW, Ste. 500, Washington, DC 20006 USA

**Keywords:** Acute care, SBIRT, Substance use, Alcohol, Tobacco, Nurse, Toolkit, Implementation

## Abstract

**Background:**

Alarming rates of unhealthy alcohol, non-prescription drug, and tobacco use highlight the preventable health risks of substance abuse and the urgent need to activate clinicians to recognize and treat risky use. Screening, brief intervention, and referral to treatment (SBIRT) is an efficacious and effective processes to identify, reduce and prevent risky use of substances. This paper describes a study protocol testing implementation of a toolkit to enhance use of SBIRT in acute care settings to recognize and address patient risky alcohol, drug, and tobacco use.

**Methods:**

This study uses a phased cluster randomized mixed method design to test nurse-led implementation of an SBIRT toolkit on one medical-surgical unit at 14 acute care hospitals (critical access, community and academic health centers). Medical surgical units will be randomly assigned to implement the SBIRT toolkit (engagement and communication, assessment, planning, training, and evaluation tools) or a wait-list usual care control group that begins implementation 6 months later. Primary endpoints are documentation of SBIRT delivery in randomly selected electronic medical records at baseline, 6 months and 12 months after group 1 implementation (61 records per unit per time period, N = 2562). Two surveys will be administered to unit nurses: smoking cessation activities will be assessed at baseline and SBIRT use will be assessed on randomly-selected days after implementation. In addition, site coordinators will complete a baseline capacity assessment, an implementation fidelity survey post-implementation, and a structured interview at the end of the study. Multilevel mixed-effects effects logistic and linear models will be used to analyze use of SBIRT and cost outcomes.

**Discussion:**

This study will guide subsequent SBIRT implementation, dissemination, and spread across rural, community and urban healthcare systems throughout the state and beyond. The long-term objective is to activate clinicians to recognize, intervene and refer people with risky substance use to improve health and decrease substance use disorders.

*Trial registration* ClinicalTrials.gov NCT03560076

## Background

According to the World Health Organization, mental health and substance use disorders will surpass all physical diseases as a major cause of disability worldwide by 2020 [1]. In the United States, the annual total estimated societal cost of substance use disorders is $510.8 billion [2]. The negative outcomes and costs associated with substance use are not exclusive to any specific substance. For example, alcohol use is linked to significant economic and health costs on a global scale [3]. It is often a contributing factor in homicides, suicides, crimes (violent, including homicide, and non-violent), motor vehicle collisions (non-fatal and fatal), and unintentional injuries to the drinker and others in his/her environment [4]. Heavy drinking can lead to a pattern of abuse and/or dependence that is often associated with other harmful behaviors such as cigarette smoking, unsafe sex, and illicit drug use [5]. Even lower levels of drinking, while potentially asymptomatic, are associated with health risks [6].

In addition, despite a steady decline in smoking rates among the general population over the past several decades, tobacco use remains the primary known preventable cause of morbidity and mortality in the United States, resulting in an estimated 480,000 deaths annually from tobacco-attributable diseases [7]. Quitting smoking has a greater impact on cardiovascular risk than changes in blood pressure, weight, physical activity, or lipids [8]. Finally, and of particular relevance to the national public health and medical infrastructure in the United States, the rate of non-medical prescription pain reliever use reported in 2014 by US residents between the ages of 18–25 was 2.8% [9]. Further, between 2004 and 2009, emergency department visits increased 98.4% in the US related to non-medical use of prescription drugs [10]. These data have meaningful implications for the US healthcare system. Over 36.5 million adults are admitted annually to acute care hospitals in the US [11]. In such settings, approximately one-third of adult patients screen positive for high-risk drinking or drug use [12].

One promising public health response to this problem is screening, brief intervention, and referral to treatment (SBIRT), which is a comprehensive, integrated approach to the delivery of early intervention and referral to treatment services for persons at risk for or with substance use disorders and/or tobacco use and dependence [13]. Rather than a specific, individual service, SBIRT is a patient care framework based on identifying risk via validated screening tools, intervening on that risk in a manner that is clinically appropriate, and referring to specialty treatment when the risk cannot be managed on-site [14]. Data from a large cross-site evaluation from diverse healthcare sites (n = 754,525) indicate that most patients who screen positive are recommended for a brief intervention (BI 68.8%), followed by brief treatment (BT 14.0%) and referral to specialty treatment (RT 17.2%) [15]. However, SBIRT screening in hospital settings tends to identify both a higher frequency of positive screens for alcohol and increased severity level of those screening scores, relative to outpatient primary care, indicating greater likelihood of patients needing referral [16, 17].

The evidence basis for SBIRT is complex and varies considerably by type of substance, activity, and severity of use. Brief interventions (BI) for unhealthy alcohol use (‘misuse’) are supported by a systematic reviews [18–20] as well as the largest SBIRT evaluation study to date [15]. Alcohol BIs have also demonstrated efficacy for underage drinkers in the ED [21]. However, alcohol BI does not appear to be efficacious for very heavy or dependent users [22]. Further, a recent meta-analysis found no evidence that BI, naturally results in engagement with alcohol-related care [23], though other researchers have disputed this claim [24]. Few SBIRT studies focus specifically on outcomes from referral to treatment (RT) for alcohol [25], sometimes due to difficulty disentangling the type of service provided [26], though the cross-site evaluation from SAMHSA’s SBIRT initiative reported a moderate/large effect for RT [15]. Due to this deficit in the literature, one research team recently conducted a proof-of-concept study for their alcohol RT, yielding promising results for patient engagement with treatment post-SBIRT [27].

SBIRT for tobacco use provides a variety of intervention types ranging from minimal interventions (brochures) to BIs, nicotine replacement therapy (e.g., nicotine patches), and quit line referral. Evidence for these components has generally supported moderate effects on tobacco abstinence, especially for BI or BI with nicotine replacement [7, 28–33]. Some of the research indicates that even minimal tobacco-centric interaction (less than a BI) may have an effect on abstinence [29]. The specific effects of any given component or mixture of components continue to be under investigation, such as in a recent (2018) 16-arm factorial study protocol of tobacco SBIRT focusing on BI, nicotine replacement, quit line referral, and smoke-free text messaging [34].

However, extant evidence supporting SBIRT for other substances is less well established. Some studies and reviews support SBIRT for other substances [14, 35–39]. However, other recent studies found no evidence of efficacy for BI for other substances [40–43], no evidence that BI for other substances reduces negative consequences of drug use [44], and no evidence that BI [43, 45] or RT [42] for other substances increases receipt of treatment. A recent meta-analysis on this subject concluded that the current evidence basis is generally insufficient to make a determination [46]. However, DiClemente’s review of reviews did find support of motivational interviewing (MI) for marijuana, while retaining the perspective of insufficient evidence for other substances (e.g., methamphetamine and opiates) [18]. A recent, innovative modification of SBIRT, called STIRT (screening, treatment initiation, and referral to treatment), included revised protocols and medication-assisted treatment for opioid dependent patients. In a randomized trial, the authors reported increased engagement in opioid treatment and abstinence at 30 days compared to traditional SBIRT and control groups [47].

The SBIRT process is a complex clinical process encompassing multiple components and activities, each of which are often not documented thoroughly, resulting in a body of literature that can be difficult to interpret. O’Donnell and colleagues identified a variety of papers pointing to a ‘lack of training or suitable intervention materials’ as one of several implementation-level barriers to SBIRT [48]. Findings in the SBIRT literature are also mixed, and differentials in implementation strategies may also account for some of the variance in SBIRT trial outcomes [18, 46]. One clear conclusion that can be drawn is that there is a need for randomized trials that thoroughly describe and test specific implementation methods. As a result, this proposed study will examine the impact SBIRT implementation on processes of care across rural, community and urban acute-care hospitals

In particular, this project challenges and seeks to shift current clinical practice paradigms within hospitals by examining the process outcomes associated with a standardized SBIRT process and workforce training toolkit for hospital nurses. Recent research has supported SBIRT training for nurses both as part of academic curricula [49, 50] and as part of quality improvement [51]. The extant patient flow within the hospital system selected for this trial has been responsive to the 2006 Institute of Medicine recommendation to coordinate mental health and substance use services with general health care [52]. But even though hospitalized patients are asked about their substance use on admission, validated screening tools are rarely used, brief interventions are often not conducted, and referrals for treatment are not completed. A hospital admission offers an opportunity for nurses to identify and intervene with patients who are currently using substances at a time when they are already seeking professional care. The health system is therefore ideal for a cluster randomized mixed method approach testing the effectiveness of a toolkit on implementation of SBIRT.

Our prospective study is innovative and promising on multiple levels. It is likely that results will provide information that will support the standardization of both the SBIRT processes and mechanisms of workforce training to address a significant behavioral health problem (substance use) that can be deployed across the health system. This includes both the consistency of inpatient hospital screening and brief intervention and advancing community partnerships for referral to treatment and assessing long-term outcomes. As observed in prior literature, many complexities in interpreting current SBIRT literature result from non-standardized implementation and effectiveness efforts. Therefore, studying implementation is a crucial aspect of the protocol. This approach: (1) utilizes a conceptual framework that maximizes facility engagement in the adoption and implementation of SBIRT; (2) creates buy-in from key stakeholders through the use of a train-the-trainer approach; (3) generates outcome data to facilitate future innovations in practice; (4) produces a SBIRT toolkit with procedures, tools, instruments, implementation guide, and resources to disseminate and sustain SBIRT use; (5) employs an interprofessional team of researchers, educators, and clinicians; and (6) facilitates the adoption of SBIRT across the health system.

Our proposed study partners with a large health system in Indiana and aligns with the State of Indiana Substance Abuse Prevention and Mental Health Promotion Strategic Plan (2012–2017) [53] by implementing SBIRT to improve the identification of people with at risk drug, alcohol or tobacco use, brief interventions and referral to treatment (when indicated). The short-term goal is to inform SBIRT implementation methods for use across rural, community and urban settings. The long-term goal is to develop an SBIRT toolkit with procedures, tools, instruments, implementation guide, and resources to disseminate and sustain SBIRT use to improve access to quality care and outcomes for people that use tobacco, non-prescription drugs, and/or drink an unhealthy amount of alcohol.

The purpose of this study is to examine SBIRT implementation methods across rural, community and urban acute hospital settings. The first aim is to test if implementation of SBIRT improves care delivery and referral of hospital inpatients currently using tobacco, alcohol or non-prescription drugs. The second aim is to evaluate the cost of SBIRT implementation and delivery for the healthcare system.

In US acute care settings, an estimated 1.8 million annual inpatient stays are primarily due to a behavioral health or substance use disorder [54], despite concerted national efforts to reduce the prevalence and incidence of these risky behaviors. There remains an urgent need to advance behavioral health care practices using sophisticated and deliberative approaches to implementation. As this section has demonstrated, the phased cluster randomized approach of SBIRT described here is the next step to advance knowledge about how best to implement SBIRT and if implementation improves use of the SBIRT process moving forward.

## Methods

### Conceptual framework

The Model for Considering the Determinants of Diffusion, Dissemination and Implementation of Innovations in Health Services Delivery and Organization guides this study [55]. This model frames what is known about the adoption of innovations within organizations and guides successful implementation of innovations. The foundation for the conceptual model is based on a narrative synthesis of theory and research that guides effective implementation of innovations in organizations. No one intervention is always effective, and interventions are likely to be more effective if based on assessment of barriers, use multiple strategies, and are system focused [55].

*Antecedents* are attributes of the hospital, such as structural aspects and leadership support that foster incorporation of new evidence, making the organization receptive to change. *Readiness* is the organization’s receptiveness to change, including support and advocacy for the change and dedicated time and resources. Antecedents and readiness are determined through an organizational assessment. *Adoption and/or assimilation* represent the process of incorporation of new evidence into practice. This study includes SBIRT training and nurse SBIRT knowledge (test) and competency (skill validation). *Implementation* is the actual use of the new evidence. In this study, implementation includes the use of SBIRT (survey randomly selected days) and smoking cessation counseling (Smoking Cessation Counseling Scale). *Consequences* are the result or outcome of adoption of new evidence. In this study, the consequences are SBIRT documentation included in the Electronic Medical Record (EMR) and patient follow-up if they are referred for treatment [55].

### Description of intervention

This study protocol will incorporate standardized education on SBIRT through a train-the-trainer approach. Registered Nurse site coordinators from each hospital will receive 8 h of training on the SBIRT process, system strategies to facilitate organizational uptake, and how to teach SBIRT to others. Study investigators will develop this training. The training will be informed by information from the Substance Abuse and Mental Health Services Administration’s public health model of universal screening, early intervention, and treatment for people who have problematic or hazardous alcohol, drug, or tobacco use and dependence, as well as content from the Indiana Prevention Resource Center and Institute for Research on Addictive Behavior. After the site coordinator training is complete, site coordinators at each facility will train additional clinical nurses in SBIRT and develop a referral process specific to their organization’s needs and resources. The site coordinators may also partner with additional staff within their hospital, including social workers and respiratory therapists, to encourage widespread adoption. The site coordinators will partner with unit leaders at each facility to decide how many additional clinical nurses will receive SBIRT training and which employees will receive training. These training interventions are reproducible, publically available, and do not require direct control from the research team. During training, the site coordinators will receive a toolkit that includes the standardized education, engagement and communication tools, assessment tools, planning tools, and evaluation tools to assist with implementation at their facility.

In addition to the toolkit and preparation of site coordinators, the investigators will use several strategies to facilitate implementation. Investigators will provide ongoing consultation to the site coordinators about SBIRT and implementation. Each month, the investigators and site coordinators will participate in a team meeting to discuss implementation barriers and facilitators. Site coordinators will be able to share tools they have developed and strategies they have used to promote implementation. Investigators will encourage adaptability of the intervention to the organizational context while preserving fidelity. The investigators may also make site visits to support implementation of SBIRT and SBIRT education. At the completion of the study, the site coordinators and investigators will work together to enhance the toolkit that was provided during training.

### Study subjects

This study will be conducted at a large healthcare system in the Midwest. Table 1 includes a description of the hospital sites, type and bed size. The hospital sample includes four community hospitals, four academic health centers, and six critical access hospitals with bed size ranges from 15 to 858 beds.

**Table 1:** Hospital characteristics by type (N = 14)

	N	Bed range	Mean beds
Academic Health Centers	4	38–858	413
Community Hospitals	4	127–375	214
Critical Access Hospitals	6	15–25	23

### Study design and sample

A phased cluster randomized mixed methods design will be used for this study. Each hospital within the healthcare system is eligible to participate, and the chief nursing officer of each hospital (N = 14) submitted a letter of support for the study. One medical surgical unit within each hospital will be selected by the nurse executive to participate. This selection will be based on the nurse executive’s assessment of the nursing unit capacity to participate in the study and implement a change in practice.

A computer-generated allocation based on a stratified random sampling approach using STATA will be used to randomize hospitals into an intervention or wait-list usual care control group. Prior to randomization, hospitals will be stratified by type (academic health center, community hospital, or critical access hospital). Allocation to the intervention or wait-list usual care control groups will be based on cluster, and participants will be notified of their cluster allocation. Participants in the control group will provide the usual care that is standard for that facility, and then the site coordinators will receive the intervention 6 months after participants in the intervention group (Table 2).

**Table 2:** Study design

	Collect pre-data	Intervention		Collect 6-month data	Collect 12-month data
Group 1 (Intervention Group)	0_1_	×		0_2_	0_3_
Group 2 (Wait-List Usual Care Control Group)	0_1_		× (6 months after Group 1)	0_2_	0_3_

All hospitals will receive the intervention. Group 1 (n = 7) hospitals will participate in the implementation intervention first, with Group 2 (n = 7) following 6 months later. Using this phased approach allows for comparisons between a control and intervention group, as well as within hospital changes longitudinally, and ensures there are no ethical concerns related to withholding the SBIRT implementation.

### Data sources

To evaluate SBIRT implementation, several data sources will be used as summarized in Table 3. To assess antecedents to change and readiness for change, the site coordinators will complete a baseline assessment of their hospital for structural capacity (e.g., buy in of stakeholders), staff capacity (e.g., staff competencies), organizational support (e.g., leadership support), technical capabilities (e.g., access to technology), and fiscal capacity (e.g., nurse staffing resources). The qualitative and quantitative baseline assessment was developed by the investigators and will be used to identify areas that need to be addressed before implementation of the study. The site coordinators and investigators will use this data to develop a facility-specific plan to address gaps prior to implementation.

**Table 3:** Conceptual framework concepts and operationalization

Concepts	Operationalized	Timing
Antecedents	Organizational attributes (SC)	Program development
Readiness	Attributes of nurses (RN)	Before implementation
Adoption	SBIRT Knowledge (SN)	Post training
	Brief Intervention Adherence and Competency Scale (observation of SN by SC)	Post training
Implementation	Smoking Cessation Counseling Scale (RN)	During implementation
	SBIRT Use and time estimates for each component (SN)	Post implementation, 5 randomly selected days
	Implementation fidelity assessment survey (SC)	Post implementation
	Monthly worksheet (SC)	Continuously
Consequences	SBIRT component documentation in medical record (electronic data abstraction and SC manual data abstraction)	Baseline, 6, and 12 months
	Time estimates for each component delivery (SN)	Post implementation, 5 randomly selected days
	Training details (e.g., number trained, meeting time, administrative time, etc.) with implementation fidelity assessment survey (SC)	Post implementation
	Barriers, facilitators, lessons learned through interviews (SC)	Post implementation End of data collection

*SC* site coordinator, *RN* All direct care nurses on study unit, *SN* all trained nurses

To assess adoption and assimilation at the completion of SBIRT training, site coordinators and direct care nurses will complete a 10-item test constructed for this study to assess knowledge about screening tools, motivational strategies, and referral tools. In addition, competency will be assessed using the Brief Intervention Adherence and Competence Scale [56]. These data will be used to evaluate training and verify SBIRT knowledge and competency.

Actual implementation will be assessed using several strategies. Each month, site coordinators will complete a qualitative and quantitative monthly worksheet developed by the investigators that includes information about the time spent on implementation, changes in care processes, barriers and facilitators to implementation, and lessons learned. On five randomly selected days, site coordinators will collect quantitative data regarding SBIRT use. After each patient admission on the randomly selected days, the direct care nurse or site coordinator will complete a survey regarding fidelity to the different components of SBIRT and the time required to complete SBIRT for that patient. Site coordinators will also complete a survey regarding implementation fidelity. This quantitative and qualitative survey includes questions about the number of nurses trained in SBIRT and actions taken to support SBIRT implementation. Finally, all direct care nurses on the study units (including nurses who did not receive training in SBIRT) will be invited to complete the Smoking Cessation Counseling Scale [57–59]. This survey assesses the extent to which clinicians comply with evidence-based smoking cessation guidelines. Data from the monthly worksheets, SBIRT use assessment, implementation fidelity assessment, and smoking cessation counseling scale will be reviewed by the investigators at the completion of data collection. This information will inform feedback to site coordinators, identify barriers and facilitators to implementation, and inform potential modifications to implementation strategies.

The consequences of SBIRT implementation include the delivery of SBIRT components and the costs of implementation. Delivery of SBIRT components will be measured by the documentation of screening, brief intervention, and referral to treatment for alcohol, drugs, and tobacco, individually and combined. These data will be based on the Joint Commission Quality Measures for alcohol, drug, and tobacco use [60]. The components of SBIRT will be documented in the medical record by healthcare providers who complete the screening, brief intervention, and referral to treatment. All hospitals use the same electronic medical record system. Sixty-one medical records at each site will be randomly selected through automated processes. Inclusion criteria are adult (≥ 18) patients admitted and discharged from units selected for participation in this study within data abstraction periods (baseline, 6 months and 12 months). De-identified data will be abstracted using an electronic data abstraction report. Automated data abstraction will be validated via comparison of electronic and manual data abstraction in a sub-sample of records at each hospital. If required, the site coordinators will conduct the medical record review manually. We will collect data regarding the number of patients screened for alcohol, tobacco, and drugs using a validated tool, and the percentage of patients who screened positive for each substance and then received a brief intervention and/or referral to treatment. The costs of implementation will be assessed using the monthly worksheets completed by the site coordinators and the SBIRT use reports. Data regarding care delivery and costs of implementation will be assessed. At the completion of the study, site coordinators will complete a semi-structured interview to describe their experiences as well as barriers and facilitators of implementation.

### Statistical analysis

A sample size calculation was conducted to determine the number of medical records needed in each of the 14 study hospitals, to achieve an 80% power at an alpha level of 0.05 to address the quantitative aims of the study while taking the clustered randomization into consideration [61, 62]. Sample size calculations were based on an intracluster correlation of 0.03. In case intracluster correlation is lower than 0.03, our statistical tests provide an even larger statistical power. For the between-group comparisons regarding the binary outcomes of Aim 1, detecting an absolute difference in a proportion between the two groups of 16% with an at least 80% power, using a Z-statistic that accounts for the intracluster correlation, requires 61 medical records per hospital. Based on this sample size for each observation time-point, we are also sufficiently powered to test for within group changes in the control group (post-intervention vs pre-intervention) since within-group comparisons are expected to have a larger power compared to between-group comparisons. Sample size calculations were conducted using the PASS 13 software.

The analysis has been a priori defined to address the research aims. Descriptive statistics will be computed for all variables to ensure data quality, identify patterns of missing and out-of-range values, and evaluate the assumptions of statistical tests. In general, missing data will be evaluated considering both descriptive statistics and possible reasons for non-response. The assumption of missing at random (MAR) [63] is evaluated based on extensive data considerations and discussions with the data collecting personnel on the reasons for nonresponse for the non-medical record data. If the MAR assumption is plausible, multiple imputation methodology will be applied [64]. If there are missing data in more than one variable, multiple imputation through chained equations will be performed [65]. Inclusion of auxiliary variables that are associated with the probability of missingness in the imputation model will be considered in order to make the MAR assumption more plausible. In this case, standard errors will be estimated through nonparametric bootstrap methods in order to avoid the consequences of uncongeniality between the imputation and analysis models on Rubin’s standard error estimator [66, 67]. If there are no variables that make the MAR assumptions plausible, a sensitivity analysis will be performed under various missing not at random scenarios to evaluate the robustness of inference against the violation of the MAR assumption. Remediation of normal distribution assumption violations will be accomplished using methods such as the Box-Cox family transformations, or other methods as appropriate. Assessment of internal consistency reliability of scales will be carried out using the Cronbach’s alpha coefficient. Analysis for each study aim will be performed using the statistical packages SAS or STATA. Any imbalances due to chance will be addressed by including the corresponding variables as covariates in the multivariable regression models for each outcome. Intention-to-treat analysis will be performed in accordance to the pragmatic trial model, in the sense that all participating units will be included in the analysis regardless of their level of adherence to SBIRT.

#### Aim 1

Evaluation of the effect of implementation strategies will be focused on the use of SBIRT. To estimate the effect of implementation (training, toolkit) of SBIRT on clinical processes, we will use multilevel mixed-effects logistic regression models to account for both the hospital unit clustering effects and the potential observation dependence within each phase of the study (i.e., baseline, 6 months, 12 months), while adjusting for variables found to be imbalanced between the study groups. Potential confounders such as organizational and nurse attributes will also be considered for inclusion in the model to avoid any residual confounding effects. Based on these models, both between and within-group effects will be estimated and model-based estimates of the effect size (i.e., odds ratio) will be calculated along with the associated 95% confidence intervals. We will additionally evaluate the intervention effect sustainability in the intervention group by comparing, using the appropriate estimate from the fitted model, the proportion of SBIRT use at 6 months versus the corresponding use at 12 months.

#### Aim 2

Evaluation of the cost of implementation and delivery of SBIRT will be focused on the system perspective. Tailored implementation of SBIRT for local healthcare settings requires appropriate budgeting and resources. Data on both human resources and tangible resource use will be measured during the current study. These data will inform future implementation and budgeting. We will estimate costs of implementation based upon phase of implementation, including the start-up, deployment, and maintenance costs of the adoption of SBIRT. Specific cost categories will collected via the monthly worksheet including personnel (training & education; deployment), site coordinator time spent on implementation, and SBIRT related expenses (e.g., written materials). Costs of SBIRT delivery will be estimated using a random sample of registered nurse’s report of estimated minutes interacting with the patient during the SBIRT process. The cost outcomes will be analyzed using mixed-effects linear regression models to account for the hospital unit clustering.

### Qualitative analysis

Post study structured phone interviews will be conducted with site coordinators. The responses to questions include pre-defined categories and a narrative, which will be documented by the investigator and verified with the participant. Quasi-statistics will be used to report the categories of responses. Narrative comments will be reviewed, coded and categorized by two independent study team members.

## Discussion

There are a number of strengths and limitations of this study related to the design, methods, and primary endpoints/outcomes. In terms of strengths, the use of a cluster randomized phased approach allows comparison of an intervention and control group, as well as within hospital differences before and after implementation of the intervention. It also allows both groups to participate in the intervention and implementation (providing data from 14 hospital unites to inform implementation guides for future use). Using a mixed method approach will provide data from multiple sources including observations, survey, interviews and secondary data from EMRs to inform the evaluation. The collection of organizational and implementation assessments will inform lessons from successful and ailed strategies with a specific focus on nurse barriers identified by Désy et al. [68]. It is also important to note that this study is being conducted at a time when the opioid epidemic is a major public health priority and health systems are seeking better processes to identify and treat people with risky substance use.

Despite these strengths there are a number of limitations of the study including use of process endpoints instead of patient outcomes, the implementation process time/skill/resource demand, and use of the EMR data. The absence of patient clinical outcomes precludes the ability to evaluate if the intervention resulted in lower substance use, treatment success or patient experience. Given the current lack of strong evidence for SBIRT on such outcomes for all substances that are being targeted (e.g., drugs), a study linking SBIRT in medical units is needed. This study provides the first step toward studies of clinical outcomes by evaluating if this evidence-based practice is usable, implementable and if patients actually receive the care they should- and how to implement the intervention. Another limitation is the time demand in learning how to use and implement SBIRT given multiple priorities for clinical nurses. While projected time estimates were shared with nurse executives, and release time from clinical duties planned and budgeted in the grant were estimated, there is a learning curve and nurse staffing can vary day to day.

Use of medical record documentation to assess SBIRT consequences may also present a threat to study validity. Compliance with processes of care captured in EMR documentation is typically used to evaluate the quality of services rendered. There is a possibility that at baseline there may be nothing documented beyond the initial screening for substance use. Even after the education, implementation may require additional time and it may take longer than 6 months for practice to change. A nurse self-report of SBIRT use on the last admitted patient on randomly selected days will be included, providing data that can be used to correlate with the EMR data.

## Conclusion

This study addresses a major public health concern—identifying, intervening, and referring people to treatment with risky substance use. A phased cluster randomized mixed method design will be used to evaluate implementation of SBIRT in acute care medical surgical unit by nurses and develop an SBIRT toolkit to enhance broad dissemination. Results of this study will affect clinical practice, health system processes and implementation and has the potential to standardize the SBIRT process enhancing action toward helping people reduce substance use risks and get treatment if appropriate- a significant innovation to current practice on medical surgical units.

## Abbreviations

BI: brief intervention
CSAT: Center for Substance Abuse Treatment
EMR: electronic medical record
IU: Indiana University
MAR: missing at random
RT: referral to treatment
SAMHSA: Substance Abuse and Mental Health Services Administration
SBIRT: screening, brief intervention, referral to treatment
